# Usage of traditional medicine during pregnancy and the associated factors among Basotho women

**DOI:** 10.4102/phcfm.v17i1.4936

**Published:** 2025-07-31

**Authors:** Lisemelo L. Chesetsi, Andrew Ross

**Affiliations:** 1 Department of Nursing and Public Health, College of Health Science, University of KwaZulu-Natal, Durban, South Africa; 2 Department of Family Medicine, Faculty of Health Sciences, University of KwaZulu-Natal, Durban, South Africa

**Keywords:** traditional medicine, prevalence, pregnancy, women, Lesotho

## Abstract

**Background:**

Many women persist in using traditional medicine despite the evidence that traditional medicines have the potential to harm both the unborn baby and the mother. Data on the extent of use of traditional medicine by women in Lesotho during pregnancy are largely unavailable.

**Aim:**

This study aimed to determine the prevalence of traditional medicine use during pregnancy among Basotho women and identify the associated factors.

**Setting:**

The study took place in Ha-Shalabeng, Ha-Molengoane and Ha-Setoko, Lesotho.

**Methods:**

A cross-sectional design was adopted, data were collected through a structured questionnaire, coded into Excel, and analysed using SPSS. Frequency distribution tables and graphs were used to describe the data on women. The χ^2^ test examined the association between categorical dependent and independent variables.

**Results:**

The prevalence of traditional medicine use during pregnancy was 40%. The factors significantly influencing traditional medicine use, included age (*p* < 0.01), educational level (*p* < 0.01), location (*p* < 0.01), transport availability (*p* < 0.04), belief in the efficacy of traditional medicine (*p* < 0.01), reasons for the type of care (*p* < 0.01) and recommendations from parents (*p* < 0.03).

**Conclusion:**

The utilisation of traditional medicine during pregnancy was found to be high. Therefore, it is crucial to have a policy in Lesotho that regulates the usage and safety of traditional medicine.

**Contribution:**

The data would be crucial in informing future research and shaping the development and implementation of traditional medicine policy, thus addressing the existing policy gap regarding traditional medicine in Lesotho.

## Introduction

The World Health Organization has conceptualised traditional medicine as:

[*T*]he sum total of the knowledge, skills and practices based on the theories, beliefs and experiences indigenous to different cultures, whether explicable or not, used in the maintenance of health as well as in the prevention, diagnosis, improvement or treatment of physical and mental illnesses.^1^

Traditional medicine has served as the basis of healthcare within households and communities for centuries, catering to approximately 70% – 80% of the global population’s primary health care needs.^1,2^ The use of traditional medicine, notably in sub-Saharan Africa (SSA), has increased over the past decades for various reasons, including the perceived shortcomings of conventional medicine in addressing emerging health conditions, such as coronavirus disease 2019 (COVID-19), human immunodeficiency virus and acquired immunodeficiency syndrome (HIV and AIDS), diabetes and cancer.^3^ Additionally, patients are increasingly seeking greater autonomy and involvement in managing their health and well-being alongside a deep-rooted cultural trust in traditional healing practices.^1,4^

Globally, scholars have identified women, including pregnant women, as the primary users of traditional medicine.^5,6,7^ The utilisation of traditional medicine among pregnant and childbearing mothers varies based on geographical location, ethnicity, cultural practices and socio-economic status.^7^ The prevalence of traditional medicine was reported to be 27% in North Europe, 11.9% in North America and 26.6% in Australia.^3^ In African countries, the use of traditional medicine among pregnant women was reported to be higher when compared to other regions, with a prevalence of up to 90%.^3^ The high prevalence of the use of traditional medicine among pregnant African women arises from the belief that it is natural and safe, adherence to cultural norms, religious affiliations and the scarcity of healthcare resources in many rural areas.^3,8^ Despite its prevalence and growing popularity, concerns regarding traditional medicine’s safety, efficacy and quality persist.^9^

While numerous studies have explored the use of traditional medicine during pregnancy and childbirth in Africa, there remains a scarcity of data regarding its prevalence in urban, peri-urban and rural areas in Lesotho. This study, therefore, aimed to establish the prevalence of traditional medicines used during and after pregnancy and to explore the factors influencing the utilisation of traditional medicine. This will offer insights that can inform research, the development and implementation of policies regarding traditional medicine use, thus addressing the existing policy gap in the country.

## Research methods and design

### Study design

The cross-sectional study explored traditional medicine use in the pre-natal and post-natal stages among women aged 18 years and older. The study design enabled the researcher to collect quantitative data from a large number of women across different health settings in the three locations.^1^

### Study area and setting

The study was conducted in three residential areas in Lesotho: Ha-Shalabeng in Maseru, the capital city; Ha-Molengoane, a peri-urban area in western Maseru; and Ha-Setoko, a rural village in the Quthing District. Ha-Shalabeng and Ha-Molengoane have community clinics, but access to healthcare remains challenging at Ha-Setoko because of the absence of a healthcare facility. These regions were purposively chosen to represent urban and rural areas and were accessible to the researcher. In keeping with factors that influence healthcare decisions, incorporating diverse levels of socio-economic development and varying degrees of access to traditional healers and healthcare services was considered important. Ha Setoko comprises 359 women, Ha-Shalabeng has 750, and Ha-Molengoane has 264 aged 18 years and older.^10^

### Study participants

The target population was women of childbearing age who had been or were pregnant, as the assumption was that they had knowledge and experience about the use of traditional medicine in the pre-natal and post-natal stages of pregnancy. The study population consisted of women aged 18 and older who resided in Ha-Setoko, Ha-Molengoane and Ha-Shalabeng and who consented to participate. Participants must have a child or children or had previously been pregnant.

### Sample size determination and sampling technique

The total study population was derived by summing the number of women over 18 years of age in all three villages, resulting in 1373 women, the assumption being that 80% would have been pregnant or had a child. The sample size for this study was determined to be 150 participants. Sample sizes for each geographic region were determined using RAOSOFT^1^ online sample size calculator according to equation 1, equation 2 and equation 3 below:


$$x=Z{\left(\frac{c}{100}\right)}^{2}r(100-r)$$ [Eqn 1]

$$n=\frac{Nx}{\left((N-1)E^{2}+x\right)}$$ [Eqn 2]

$$E=\sqrt{\frac{(N-n)x}{n(N-1)}}$$ [Eqn 3]

where: *χ* is a measure of response rate, *n* is the sample size, *N* is the population size, **r** is the fraction of responses that you are interested in, *Z*(*c*/100) is the critical value for the confidence level *c*, and *E* is the margin of error. Using a value of 7.56% for the margin of error, a 95% confidence level corresponding to an Z-score of 1.96, a population size (*N*) of 1373 and an assumed response distribution (*p*) of 50%, a sample size of 150 was determined for the survey respondents. To ensure proportional representation, the sample was stratified according to the number of eligible women in each geographic region using equation 4 below:


$$\frac{\text{Number of women in the stratum x desired sample }(150)}{\text{Total number of women in the population }(1373)}$$ [Eqn 4]

Table 1 provides the proportional distribution and sample sizes for the three geographic regions.

**TABLE 1:** Proportional distribution and sample size calculation using RAOSOFT.

Sampling unit	Population size	Sample size
Rural Ha-Setoko	359	359 × 150/1373 = 39 women
Urban Ha-Shalabeng	750	750 × 150/1373 = 82 women
Peri-urban Ha-Molengoane	264	264 × 150/1373 = 29 women

Note: Population size for each village was obtained from ‘ 2016 Census report [10].

A simple, random probability sampling technique was used to identify participants from each stratum based on a list of women who met the inclusion criteria from the village chiefs.

Each name on the list was assigned a unique number from the 1^st^ to the N^th^ position, with a random number picked until the required sample size was achieved.

The principal researcher is a Mosotho woman, born and raised in Lesotho, who contacted the women on the list and asked if they would be willing to complete the questionnaire, with all respondents selected from the list agreeing to participate.

Because of a small sample size of the peri-urban Ha-Molengoane participants (*n* = 29), they were combined with urban participants of Ha-Shalabeng (*n* = 82) to meet the requirements for statistical power. Peri-urban participants were considered closer to urban than to rural women as Ha-Molengoane is located on the outskirts of Maseru, approximately 40 km from the city.^11^ Its position as an urban transition zone and having a clinic provided the rationale for analysing them together. This integration facilitated a more robust statistical analysis, which would not have been feasible with smaller, separate data sets.

### Data collection instrument and procedure

Face-to-face interviews were conducted by the researcher from November to December 2022, with data collected using a structured questionnaire that was designed in English and translated into the local language, Sesotho. The four sections consisted of questions relating to their socio-demographic details, usage of traditional medicine, factors influencing the use of traditional medicine, traditional medicines used during pregnancy and side effects experienced.

To maintain data quality, the questionnaire was pre-tested on 10 women from Roma community, a peri-urban community on the outskirts of Maseru, with characteristics similar to those of the intended study participants. Based on the insights obtained from this pre-testing, minor amendments were made to the questionnaire before the data collection began. The questionnaires were all reviewed and checked for completeness and consistency of data.

### Data analysis

The data on the forms were coded into Microsoft Excel and then exported to IBM Statistical Package for Social Science (SPSS) version 28 for analysis, with descriptive statistics presented as frequencies and percentages. The χ^2^ test was used to examine the association between categorical dependent and independent variables, the significance level being 0.05%.

### Ethical considerations

Ethical clearance to conduct this study was obtained from the University of KwaZulu-Natal, Humanities and Social Sciences Research Ethics Committee (HSSREC) (No. HSSREC/00004919/2022). Permission to conduct the interviews with the women residing in the specified study sites was obtained from the respective village chiefs. The comprehensive informed consent addressed all the relevant ethical considerations, such as confidentiality, data protection, privacy, and the participant’s right to withdraw from the study at any point during the interview.

## Results

### Socio-demographic characteristics

Table 3, shows all the 150 women who were invited to participate, with the majority 111 (74%) residing in urban (Ha-Shalabeng) and peri-urban areas (Ha-Molengoane), while 39 (26%) lived in a rural area (Ha-Setoko). Most participants were married (94; 62.7%), aged 40 and above (90; 60%,), had completed high school (94; 62.7%), earned less than M500 a month (89; 59.3%) and were unemployed (92; 62%).

### Prevalence of traditional medicine use

The data analysis revealed that the majority of survey participants, that is, 90 (60%) indicated that they did not utilise traditional medicine. This means that the prevalence of traditional medicine use is 60 (40%) among women, with its use being an important part of their healthcare practices.

### Factors associated with the use of traditional medicine

Table 2 presents various factors influencing the use of traditional medicine during pregnancy, with statistical analysis exploring associations between multiple factors. Marital status did not significantly impact traditional medicine utilisation during pregnancy (χ^2^ = 0.14, *p* = 0.7), with unmarried women reporting the highest use, 24 (43%), than married participants, 36 (38%). Conversely, age significantly affected traditional medicine use during pregnancy (χ^2^ = 8.36, *p* < 0.01), with those 18–40 years being less likely to utilise traditional medicine (15; 25%) than those aged 40 and above (45; 50%). Individuals with higher education levels reported lower usage rates (9; 24%), whereas those with lower education levels, particularly those with primary education, indicated higher utilisation (31; 55%). The educational level of participants was found to significantly influence the use of traditional medicine (χ^2^ = 9.86, *p* < 0.01). While their employment status did not affect the use of traditional medicine (χ^2^ = 0.62, *p* 0.43), unemployed participants demonstrated higher use (40; 43%). Similarly, household income was not a significant factor influencing use (χ^2^ = 0.97, *p* = 0.33), with those earning M500/month (*equivalent to R 500/month) or less reporting the highest utilisation rate, 39 (44%).

**TABLE 2:** Factors associated with traditional medicine use during pregnancy.

Variable	Characteristics	Use of traditional medicines				Chi-square	*p*
		Yes		No			
		No.	%	No.	%		
Marital status	Married	36	38	58	62	0.14	0.70
	Unmarried	24	43	32	57		
Age (years)	18–39	15	25	45	75	8.36	0.01*
	≥ 40	45	50	45	50		
Educational level	Primary	31	55	25	45	9.86	0.01*
	Secondary	20	35	37	65		
	Tertiary	9	24	28	76		
Employment status	Employed	20	35	37	65	0.62	0.43
	Not employed	40	43	53	57		
Household income	≥ M501	21	34	40	66	0.97	0.33
	≤ M500	39	44	50	56		
Location	Rural	23	57	17	42	6.00	0.01*
	Urban	37	34	73	66		
Distance to the nearest clinic	5 km – 10 km (*n* = 112)	45	40	67	60	0.00	1.00
	> 10 km (*n* = 38)	15	39	23	61		
Transport available (to the clinic)	No	18	58	13	42	4.41	0.04*
	Yes	42	35	77	65		
Belief in the efficacy of TM	Agree	8	44	10	56	75.11	0.01*
	Strongly agree	47	81	11	19		
	Disagree	3	8	35	92		
	Strongly disagree	2	7	25	93		
	Missing	0	0	9	100		
Reasons for the type of care (could select more than one option)	Closer to home	15	71	6	29	44.10	0.01*
	Cultural reasons	18	95	1	5		
	Good health care services	5	29	12	71		
	Health and safety	21	23	70	77		
	Less costly	1	50	1	50		
Who recommend you (could select more than one option)	Husband	13	46	15	54	12.15	0.03*
	In-laws	4	50	4	50		
	Others	0	0	1	100		
	Parents	21	60	14	40		
	Relatives	2	40	3	60		
	Self	20	27	53	73		

Note: Not married includes divorced, widowed, single and living together; M refers to Maloti (the currency of the Kingdom of Lesotho).

TM, traditional medicine; No., number.

* , Statistically significant.

The findings indicated that using traditional medicine differs significantly by geographic location (χ^2^ = 6, *p* < 0.01), with participants from rural areas reporting the highest usage rate 23 (57%) and highlighting the influence of geographical context on adopting traditional healthcare practices. Traditional medicine use was highest among women who travelled 5 km – 10 km to reach the nearest healthcare facility 45 (40%), although the distance travelled did not significantly impact its use (χ^2^ = 1, *p* = 1). In contrast, access to transport significantly influenced traditional medicine use (χ^2^ = 4.41, *p* < 0.04), with the women without the means to travel being more likely to use traditional medicine 18 (58%). The belief in the efficacy of traditional medicine significantly influenced its use (χ^2^ = 75.11, *p* < 0.01), with 47 (81%) of those who used it agreeing that it was effective. The study highlighted several reasons for using traditional medicine, with cultural practices being the most commonly reported factor, cited by 18 (95%), and significantly impacting its use (χ^2^ = 44.1, *p* < 0.01). Lastly, parental recommendations significantly influenced its use (χ^2^ = 12.5, *p* < 0.03), with 21 (60%) reporting that they used traditional medicine based on their parents’ advice.

### Traditional medicines used during pregnancy

**FIGURE 1:**
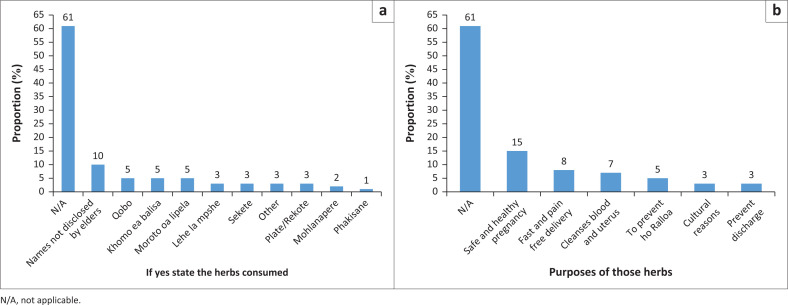
Traditional medicines used and their purposes. (a) Herbs consumed; (b) Purpose of herbs.

Among women who used traditional medicine, the most reported herbs were Qobo, Khomo ea balisa and Moroto oa lipela, each with 5% of survey respondents using them. Other herbs mentioned included Lehe la mpshe (3%), Sekete (3%), Plate or Rekote (3%), Mohlanapere (2%) and Phakisane (1%), with varying percentages of usage. Based on the results shown in Figure 1, most participants (60%) did not use traditional medicines.

The most common purpose of traditional medicines was ensuring a safe and healthy pregnancy (15%), followed by facilitating a fast and painless delivery (8%). Other reasons included cleansing the blood and uterus (7%), preventing ho Ralloa [spiritual curses], cultural beliefs (3%) and preventing discharge (3%). This suggests that traditional medicine is primarily used for reproductive health, focusing on maternal and foetal well-being.

### Adverse events profile

Among the women who reported using traditional medicine during pregnancy (40%), only 2% reported to have experienced adverse effects. The primary concern was that they took longer to deliver. In contrast, most participants (36%) did not experience any adverse effects. This suggests that for most participants, the use of traditional medicine did not pose any significant health challenges, although a small minority experienced delays in childbirth delivery.

## Discussion

The study aimed to determine the prevalence of traditional medicine use, explore the factors associated with use and the traditional medicines used during pregnancy. The prevalence of traditional medicine use during pregnancy among Basotho women in this study was 40%, which is higher than the 19.7% reported in Central Ethiopia and 10.9% in Tanzania.^12,13^ However, this result is lower than that of similar studies conducted in Nigeria, Uganda and Zimbabwe.^14,15,16^ Prevalence rates may differ because of cultural preferences and differences in access to healthcare services and traditional medicine practitioners.

The use of traditional medicine was notably higher among unmarried than married women, which differs from a Nigerian study that reported a higher prevalence of traditional medicine use among married women.^17^ While no association was noted between marital status and the use of traditional medicine during pregnancy (*p* = 0.7), a significant association was found between participants’ age and its use (*p* < 0.01), which was higher among women aged 40 and above, as was reported in a study from Zimbabwe.^18^ This finding may be attributed to participants drawing on their experiences with traditional medicine use, mainly when they were pregnant, and healthcare services were less developed or less accessible, leading them to rely more on traditional remedies during pregnancy.^12,19^

Table 3, shows all the 150 women who were invited to participate, with the majority 111 (74%) residing in urban (Ha-Shalabeng) and peri-urban areas (Ha-Molengoane), while 39 (26%) lived in a rural area (Ha-Setoko).

**TABLE 3:** Socio-demographic details.

Variable	Characteristic	No.	%
Location type	Urban and peri-urban	111	74.0
	Rural	39	26.0
Marital status	Married	94	62.7
	Not married	56	37.3
Age (years)	18–39	60	40.0
	≥ 40	90	60.0
Educational level	Primary	56	37.3
	High school	57	38.0
	Tertiary	37	24.7
Household income	≤ M500	89	59.3
	≥ M501	61	40.6
Employment status	Employed	57	38.0
	Not employed	93	62.0

**Total**		**150**	**100.0**

Note: M refers to Maloti (the currency of the kingdom of Lesotho).

No., number.

The participants’ educational level was associated with the use of traditional medicine, with those having primary education showing the highest likelihood, while those with tertiary education had the least likelihood of its use. While this has been reported in several studies,^3,7,20,21^ research conducted in Uganda reported a higher usage rate among women with tertiary education.^22^

The study results also revealed that the use of traditional medicine in pregnancy was higher among unemployed women and those from the lowest-income households, this being similar to a systematic review by James et al.^23^ No association was noted between employment or household income and the use of traditional medicine. However, a significant association was noted between the use of traditional medicine and place of residence, with rural participants demonstrating higher utilisation rates than their urban counterparts, as was reported in a study conducted in Ethiopia.^24^ The higher prevalence in rural areas, particularly in Ha-Setoko, may be attributed to the limited availability of healthcare facilities, the lack of transport and the greater accessibility of traditional healers in this region.

This study observed that women who lived within 5 km – 10 km of health facilities were among the highest traditional medicine users. In contrast, those travelling longer distances were least likely to utilise traditional medicine. This result contradicts findings from a study conducted in Uganda, where the prevalence was higher among women travelling longer distances to the nearest clinic.^25^ No significant association was noted between the distance travelled to the closest healthcare facility and the use of traditional medicine. Women without access to transport were also most likely to use traditional medicine, as was reported in a study conducted in Uganda.^25^

The findings revealed that women who used traditional medicine during pregnancy were influenced by the belief in its efficacy, comparable to a survey conducted in Nigeria, which reported its use by approximately 43% of women.^14^ The perception of the effectiveness of traditional medicine in solving pregnancy or health complications tends to influence their continued use.^15^ There was also a significant positive association between the use of traditional medicine and cultural practices, being one of the drivers of its use during pregnancy, with similar results being found in studies conducted in South Africa and Tanzania.^26,27^ Similar to a study conducted in Zimbabwe, a positive association was noted between traditional medicine usage and information received from their parents.^28^

The participants’ most commonly used traditional medicines were Qobo, Khomo ea balisa, Moroto oa lipela, Lehe la mpshe, Sekete, Plate or Rekote, Mohlanapere and Phakisane. This finding is consistent with the results of a quantitative study conducted in Lesotho, which indicates that traditional herbs such as qobo, phakisane and khomo ea balisa play an essential role in treating reproductive problems and in the lives of pregnant women.^29^ However, different traditional remedies were reported in a study conducted in KwaZulu-Natal Province, South Africa.^8^ Variations of traditional remedies used across countries may be because of differences in cultural traditions and vegetation types.

Participants stated that they utilised these remedies to ensure a safe and healthy pregnancy, facilitate a fast and painless delivery, cleanse the blood and uterus, support cultural beliefs, prevent ho Ralloa [spiritual curses] and vaginal discharge. In contrast, a study conducted in Uganda identified different reasons for using traditional medicine and included treating symptoms such as fever, abdominal and waist pain, respiratory illnesses and skin problems.^30^ This suggests that traditional medicines address various ailments based on the patient’s condition or health needs. Most participants who used traditional medicines did not specify their reasons or purposes for using traditional herbs, which could be associated with the secrecy many traditional healers and African elders maintain regarding traditional medicine and their intended purposes. Consequently, participants may lack information as it was not disclosed to them.^11^ In this study, adverse effects among pregnant women were reported by only 2% of the participants who used traditional medicine; other researchers have documented similar findings and highlighted concerns about possible negatives and the toxicity of such formulations.^8,28^

### Limitations, strengths and recommendations

This study was subject to certain limitations, including the small sample size. It was confined to only two of the country’s 10 districts and may not reflect the opinions of women living elsewhere. Recall bias may pose a limitation, as some participants may not accurately remember the traditional medicines they used during past pregnancies, potentially influencing the results, as is correct reporting, with some women possibly not indicating their use of traditional medicine. While acknowledging the study’s limitations, the insights obtained hold significant value in understanding the factors influencing the use of traditional medicine and the prevalence of its use during and after pregnancy.

We recommend further research that expands beyond the two districts in Lesotho and includes different communities in urban, peri-urban and rural areas. In addition, the researchers recommend additional studies with different methodologies and larger sample sizes, which will provide a more comprehensive understanding of the influencing factors and the prevalence of use of traditional medicine in Lesotho, thus informing strategies or policies for the effective integration of traditional medicine in the healthcare system of Lesotho.

## Conclusion

The prevalence of traditional medicine use during pregnancy was found to be notably high, with age and educational level significantly influencing its use during pregnancy among participants. Several traditional medicines were reported, with their specific purposes, such as promoting safe and healthy child delivery, with culture and access to traditional healers being given as reasons for their continued reliance on such services. These findings underscore the significant role that traditional medicine plays in pregnancy and highlight the need for research and establishing a traditional medicine policy in Lesotho to assess traditional medicine safety, efficacy and usage in alignment with World Health Organization recommendations.

## Footnotes

1 Sample size calculation (http://www.raosoft.com/samplesize.html).
